# The discriminatory ability of the body roundness index and body mass index for metabolic diseases in Korean adults: a comparative study

**DOI:** 10.4178/epih.e2025069

**Published:** 2025-12-09

**Authors:** Soo Jeong Yoon, Ho-Jang Kwon

**Affiliations:** 1 Total Healthcare Center, Kangbuk Samsung Hospital, Sungkyunkwan University School of Medicine, Seoul, Korea; 2 Department of Preventive Medicine, Dankook University College of Medicine, Cheonan, Korea

**Keywords:** Body mass index, Body roundness index, ROC curve, Metabolic diseases

## Abstract

**OBJECTIVES:**

Obesity is a major risk factor for metabolic diseases; however, body mass index (BMI), the most widely used anthropometric indicator, inadequately reflects fat distribution. The body roundness index (BRI) has been proposed as a more precise measure of abdominal obesity.

**METHODS:**

Data from the 2007-2022 Korea National Health and Nutrition Examination Survey (KNHANES) were used. Discrimination for diabetes, hypertension, hypercholesterolemia, and metabolic syndrome was assessed by calculating the area under the receiver operating characteristic curve (AUROC) and estimating odds ratios (ORs).

**RESULTS:**

AUROC values ranged from 0.739 to 0.844 for BMI and 0.745 to 0.851 for BRI, with both indices demonstrating their highest performance for metabolic syndrome. BRI outperformed BMI for 3 metabolic diseases except hypertension, with the largest AUROC difference observed for diabetes (0.01). Quintile-based ORs showed stronger associations for BRI, indicating approximately 2-fold higher risks for diabetes and metabolic syndrome compared with BMI. Subgroup analyses identified the most pronounced differences for diabetes in female aged 45 years or older and for metabolic syndrome in male aged 45 years or older. For both indices, the risk associated with increasing quintiles was greater in the younger age group, especially among female under 45 years, in whom the risk of metabolic syndrome was markedly higher in the highest BRI quintile compared with the lowest quintile.

**CONCLUSIONS:**

BRI showed superior discriminatory power and stronger associations with metabolic diseases compared with BMI, suggesting that it may complement BMI as a useful screening indicator in clinical and public health settings.

## GRAPHICAL ABSTRACT

[Fig f4-epih-47-e2025069]

## Key Message

Using data from the 2007-2022 Korea National Health and Nutrition Examination Survey, this study compared the discriminatory performance and associations of the body roundness index (BRI) and body mass index (BMI) with metabolic diseases and found that, overall, BRI demonstrated superior discriminatory ability and stronger associations than BMI. The advantage of BRI over BMI was most pronounced for diabetes in female aged 45 years or older and for metabolic syndrome in male aged 45 years or older. Therefore, BRI may serve as a complementary indicator to BMI, providing additional value for identifying high-risk groups for metabolic diseases in clinical and public health settings.

## INTRODUCTION

Overweight and obesity pose serious health risks. As of 2021, approximately 3.71 million deaths were attributed to overweight and obesity, resulting in 129 million disability-adjusted life years worldwide [1]. If the current trend continues, it is projected that by 2050, the number of overweight or obese adults will reach 3.8 billion, which would represent more than half of the estimated global adult population [2]. Metabolic syndrome, characterized by abdominal obesity, dyslipidemia, impaired glucose tolerance, and elevated blood pressure, is also becoming more common globally [3]. These metabolic disorders are associated with elevated mortality and impose substantial societal impacts [4,5]. Therefore, accurate screening criteria and early diagnosis are essential for their effective prevention and management.

The body mass index (BMI), which has long been the most commonly used measure of obesity, is calculated using weight and height. However, it does not differentiate fat from muscle mass or reflect fat distribution, which limits its usefulness in disease screening [6]. Even individuals with the same height, weight, and BMI may vary considerably in fat distribution. In fact, mortality is more closely linked with the ratio of visceral to subcutaneous fat than with overall adiposity [7].

Therefore, there is a growing need for new anthropometric indices that can complement or replace BMI. Recently, alternative measures such as a body shape index and the body roundness index (BRI) have been developed, and research examining their utility is ongoing [8-10].

BRI was developed by Thomas et al. [11] in 2013 based on fat mass measured by dual-energy X-ray absorptiometry and visceral fat measured by magnetic resonance imaging, along with waist circumference and height. Its ability to identify individuals at increased disease risk is being actively explored. Several studies have shown that BRI demonstrates superior screening performance compared with traditional indices such as BMI, waist circumference, and waist-to-height ratio, particularly for metabolic diseases, including metabolic syndrome [12,13].

The usefulness of BRI as a disease prediction indicator has also been confirmed in cohort studies. One study evaluating the association between BRI and mortality identified a U-shaped curve similar to that observed for BMI [14]. In addition, Yang et al. [15] reported an increased risk of cardiovascular disease according to BRI trajectory, suggesting that BRI may serve as a novel indicator for cardiovascular disease risk.

Although BRI better reflects visceral fat, which is more harmful to health than BMI, further rigorous validation is necessary before it can replace BMI, especially since current medical systems, including pediatric growth charts and obesity guidelines, continue to rely heavily on BMI. Additional research is needed to evaluate its predictive value across diverse health outcomes in populations of different ages, sexes, races, and socioeconomic backgrounds.

In this context, Korea provides an ideal setting for such validation, as it conducts annual, nationally representative health and nutrition surveys that offer high-quality data to assess the clinical utility of BRI.

This study aimed to evaluate the potential of BRI to complement BMI by comparing their diagnostic performance for identifying high-risk groups for metabolic diseases in Korean adults, using data from the Korea National Health and Nutrition Examination Survey (KNHANES).

## MATERIALS AND METHODS

### Study population

This study used data from the KNHANES from 2007 to 2022. KNHANES is a nationwide, representative, cross-sectional survey conducted annually by the Korea Disease Control and Prevention Agency (KDCA) since 1998 in accordance with the National Health Promotion Act. It evaluates the health and nutritional status of Koreans through 3 components: health interviews, health examinations, and nutritional surveys. The survey collects information on socioeconomic status, health behaviors, quality of life, medical service utilization, physical measurements, clinical indicators, and dietary intake [16].

**Figure 1.**
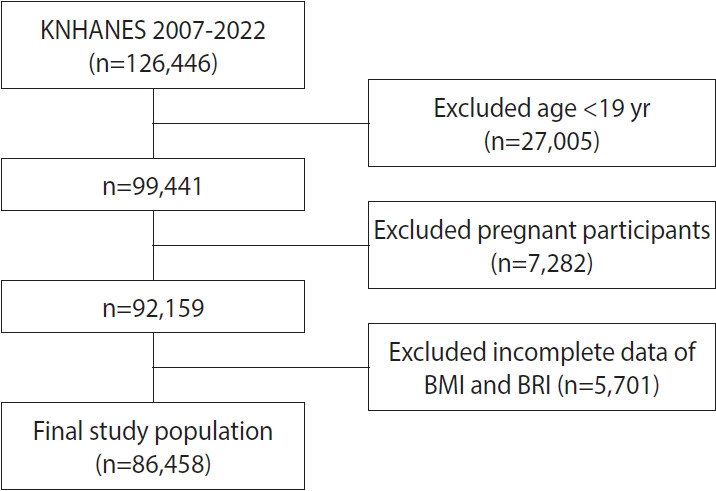
Flowchart of participant selection. KNHANES, Korea National Health and Nutrition Examination Surveys; BMI, body mass index; BRI, body roundness index.

The study sample consisted of 126,446 survey participants (4th period: 2007-2009, 24,871 participants; 5th period: 2010-2012, 25,534 participants; 6th period: 2013-2015, 22,948 participants; 7th period: 2016-2018, 24,269 participants; 8th period: 2019-2021, 22,559 participants; 9th cohort: 2022, 6,265 participants). Among the 126,446 individuals, we excluded 27,005 participants younger than 19 years, 7,282 pregnant female, and 5,701 participants with missing BMI or BRI data. The final analytic sample consisted of 86,458 adults (Figure 1).

### Analysis methods

#### BRI definition

BRI was calculated by the formula developed by Thomas et al. [11]


$$\text{BRI=364.2−365.5}\times \surd (\text{1-[waist circumference in centimeters/2}\pi {\text{]}}^{2}/{\left[0.5\times \text{height in centimeters}\right]}^{2})$$

#### Definition of metabolic diseases

The definitions of diabetes, hypertension, and hypercholesterolemia followed the official KNHANES guidelines. Diabetes was defined as a fasting plasma glucose level ≥126 mg/dL, or the use of antidiabetic medication, or a previous physician diagnosis. Hypertension was defined as a systolic blood pressure ≥140 mmHg, or a diastolic blood pressure ≥90 mmHg, or the use of antihypertensive medication. Hypercholesterolemia was defined as a fasting total cholesterol level ≥240 mg/dL or the use of cholesterol-lowering medication.

Metabolic syndrome was defined as the presence of 3 or more of the following 5 components, based on the criteria of the KDCA, which reference the scientific statement of the American Heart Association (AHA) and the National Heart, Lung, and Blood Institute (NHLBI) [17]: abdominal obesity (waist circumference ≥90 cm in male, ≥85 cm in female); elevated blood pressure (≥130/85 mmHg or the use of antihypertensive medication); elevated fasting blood glucose (fasting plasma glucose ≥100 mg/dL or the use of antidiabetic medication); reduced high-density lipoprotein cholesterol (<40 mg/dL in male, <50 mg/dL in female or the use of lipid-lowering medication); and elevated triglycerides (≥150 mg/dL or the use of medication for hypertriglyceridemia).

### Statistical analysis

BMI and BRI were compared using the t-test, analysis of variance, and the chi-square test according to demographic characteristics, including sex; age group (under 45, 45-65, over 65); education level (less than high school, college or above); household income (less than 2 million, 2-5 million, and more than 5 million Korean won); smoking status (never, past smoker, current smoker); alcohol consumption (<1 drink/mo, 1-4 drinks/mo, ≥2 drinks/wk); and physical activity (≥600 vs. <600 MET-min/wk). MET-min/wk was calculated following World Health Organization guidelines, applying 3.3 METs for walking, 4.0 METs for moderate-intensity activity, and 8.0 METs for vigorous-intensity activity [18].

Receiver operating characteristic (ROC) analyses were performed to compare the discriminatory ability of BMI and BRI for each metabolic disease. All ROC models were based on multivariable logistic regression analyses adjusted for age, sex, education level, household income, physical activity, smoking status, and alcohol consumption. Age was modeled as a continuous variable to more precisely capture its gradient effect across the study population.

We compared the associations of BRI and BMI with metabolic diseases. BMI and BRI were categorized into 5 groups based on the 20th percentile, 40th percentile, 60th percentile, and 80th percentile. Logistic regression analysis was conducted to calculate odds ratios (ORs) for each higher group relative to the lowest group, adjusting for age (continuous), sex, education level, household income, physical activity, smoking status, and alcohol consumption.

The study population was stratified into 6 groups according to sex and age group. Within each group, AUROC values and ORs for BMI and BRI were analyzed for 4 metabolic diseases. All analyses incorporated sampling weights, clusters, and strata to account for the complex survey design of KNHANES, except for ROC analyses, as the purpose of ROC analysis was to compare the discriminatory ability of BMI and BRI rather than to estimate population-level parameters. Statistical analyses were performed using Stata/SE version 19.0 (StataCorp., College Station, TX, USA).

### Ethics statement

This study was deemed exempt from ethical review and informed consent by the Institutional Review Board of Dankook University (approval No. 2024-12-002-001), owing to the use of deidentified publicly available data.

## RESULTS

### General characteristics, body mass index and body roundness index status of the study population

The mean BMI and BRI in the total study population were 23.9 and 3.42, respectively. As age increased, BRI continued to rise, whereas BMI initially increased but then declined in the oldest age group (≥65 years). Regarding sex differences, BMI was significantly higher in male, while no significant difference was observed for BRI. Both BMI and BRI showed inverse associations with socioeconomic status, demonstrating lower values in groups with higher educational attainment and decreasing trends as household income increased. For physical activity, participants engaging in ≥600 MET-minutes of exercise per week had significantly lower BRI values than those exercising less than 600 MET-min/wk, whereas BMI did not differ significantly by activity level. In relation to smoking status, both BMI and BRI were lowest among never smokers. With respect to alcohol consumption, BMI was highest in the group reporting the greatest alcohol intake (more than 2 drinks/wk), whereas BRI values were highest in the group with the lowest consumption (less than 1 drink/mo).

The prevalence of metabolic diseases in the study population was 10.0% for diabetes, 25.9% for hypertension, 16.8% for hypercholesterolemia, and 27.0% for metabolic syndrome. For all 4 conditions, both BMI and BRI were significantly higher in the disease group compared with the non-disease group (Table 1).

### Area under the receiver operating characteristic curves of body mass index and body roundness index for metabolic disease

**Figure 2.**
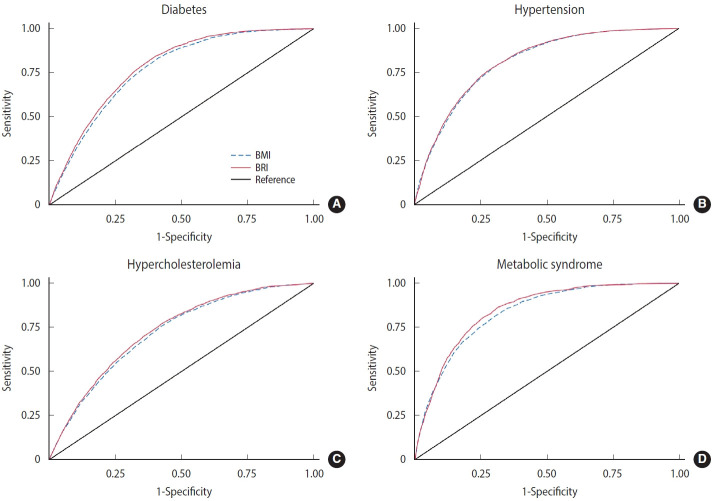
Receiver operating characteristic curves of BMI and BRI to identify subjects with (A) diabetes, (B) hypertension, (C) hypercholesterolemia, and (D) metabolic syndrome. BMI, body mass index; BRI, body roundness index.

In the total study population, the AUROC values of BRI were higher than those of BMI for 3 metabolic diseases, except hypertension (Figure 2). The AUROC values of BMI and BRI for diabetes, hypertension, hypercholesterolemia, and metabolic syndrome ranged from 0.739 to 0.844 and from 0.745 to 0.851, respectively. For both indices, the highest AUROC was observed for metabolic syndrome, and the lowest for hypercholesterolemia. The AUROC values of BRI were significantly higher than those of BMI for diabetes and metabolic syndrome, whereas no significant differences were found for hypertension and hypercholesterolemia. The largest difference between the 2 indices was observed for diabetes (0.010).

In sex-specific analyses of AUROC for BRI and BMI, all 4 metabolic diseases showed higher values in female than in male. The difference between BRI and BMI was greatest for metabolic syndrome in male (0.015), whereas in female, the largest difference was observed for diabetes (0.011).

When the AUROC values of BMI and BRI were compared by sex and age, BRI demonstrated higher values than BMI in most subgroups for all metabolic diseases except hypertension. The most pronounced differences between the 2 indices were noted for diabetes in female aged 45 years or older and for metabolic syndrome in male aged 45 years or older (Table 2).

### Body mass index and body roundness index odds ratios by metabolic disease

**Figure 3.**
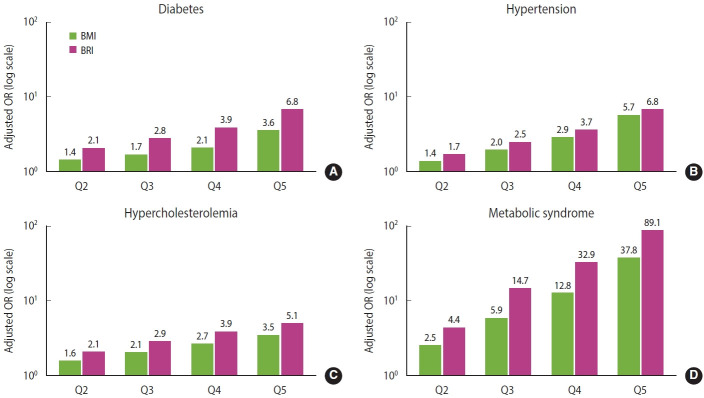
Risk of higher quintiles versus the lowest quintile of BMI and BRI for each metabolic disease (A) diabetes, (B) hypertension, (C) hypercholesterolemia, and (D) metabolic syndrome. Odds ratios (ORs) are adjusted for age, sex, education level, income, physical activity, alcohol, and smoking. BMI, body mass index; BRI, body roundness index. Quintile (Q) mean values for BMI were 19.3 (Q1), 21.8 (Q2), 23.6 (Q3), 25.4 (Q4), and 29.1 (Q5), and for BRI were 1.93 (Q1), 2.69 (Q2), 3.29 (Q3), 3.94 (Q4), and 5.27 (Q5).

ORs for each metabolic disease were calculated for the higher quintile groups of BMI and BRI, using the lowest quintile as the reference. The models were adjusted for age, sex, edcation level, household income, physical activity, smoking status, and alcohol consumption. Both indices demonstrated clear dose-response relationships, with ORs for all metabolic diseases increasing steadily across higher quintiles, and this pattern remained consistent regardless of sex or age (Figure 3).

Compared with the lowest quintile group, the highest quintile group showed significantly elevated risks of metabolic diseases, with ORs ranging from 3.5 to 37.8 for BMI and from 5.1 to 89.1 for BRI. Among the outcomes, metabolic syndrome showed the greatest increase. For all conditions, ORs for BRI exceeded those for BMI, with the largest differences observed for diabetes and metabolic syndrome, in which ORs were approximately 1.9-fold and 2.4-fold higher, respectively.

In the sex-specific analysis, BMI showed higher ORs in male than in female for all conditions except diabetes, whereas BRI showed higher ORs in female than in male for all conditions except hypercholesterolemia.

For all metabolic diseases, the ORs of both BMI and BRI were highest in the younger age groups and decreased with increasing age, and this pattern was consistent in both male and female (Table 3).

## DISCUSSION

In this nationally representative study, we compared the discriminatory performance of BMI and BRI for 4 major metabolic diseases: diabetes, hypertension, hypercholesterolemia, and metabolic syndrome. BRI demonstrated better discriminatory ability than BMI for all metabolic diseases except hypertension, with the greatest difference observed for diabetes. In the subgroup analysis, the superiority of BRI was most prominent for diabetes in female aged 45 years or older and for metabolic syndrome in male aged 45 years or older. Additionally, logistic regression analysis showed that the highest quintiles of BRI were associated with substantially greater risks of metabolic diseases compared with BMI, with differences of approximately 2-fold in magnitude for diabetes and metabolic syndrome.

Our findings are consistent with previous international research. Tian et al. [12], using data from the 2008-2009 China Health and Nutrition Survey, analyzed AUROC values for several metabolic diseases and found that BRI demonstrated superior discriminatory ability compared with BMI for all metabolic conditions (hypertension, diabetes, metabolic syndrome, and dyslipidemia) in female, and for hypertension and diabetes in male. Chang et al. [19] similarly reported that for diabetes, BRI showed higher AUROC values and ORs compared with BMI, indicating that BRI has superior discrimination power for the disease among rural populations in northeast China. Ramírez-Vélez et al. [20] reported that BRI demonstrated a higher AUROC than BMI in Colombian adults aged 65 years and older, suggesting its suitability for screening metabolic diseases in older adults. Wu et al. [21] also found that BRI was strongly associated with cardiovascular disease in younger individuals aged 55 years and below. In the Korean context, an analysis based on the Korean National Health Insurance Service (NHIS) reported that waist circumference, as a measure of abdominal obesity, was associated with increased risks of type 2 diabetes, hypertension, and myocardial infarction, and that these risks further increased with higher BMI values [22].

Our study extends this evidence by demonstrating that BRI, which integrates both height and waist circumference into a single continuous index, provides added discriminatory value beyond BMI in the Korean adult population. Using large-scale, nationally representative data spanning multiple survey waves (2007-2022), we further highlight the population-specific relevance of BRI, particularly in Asians who tend to exhibit higher body fat and visceral adiposity at lower BMI levels compared with Western populations. These findings underscore the potential of BRI as a more comprehensive and standardized anthropometric index for assessing metabolic risk in relatively lean Asian populations.

Not all studies, however, have consistently identified BRI as a superior indicator compared with BMI. Maessen et al. [23] reported that although BRI demonstrated discriminatory ability for cardiovascular diseases, it was not superior to BMI as an anthropometric measure. Similarly, Suliga et al. [24] found that for metabolic syndrome, BMI performed better than BRI in male. Thus, diverse and occasionally conflicting results have been reported for these newer indices. These discrepancies may be explained by differences in study populations, variations in cutoff values, and heterogeneity in disease outcomes assessed, suggesting that the relative performance of BRI and BMI may depend heavily on population-specific and methodological factors.

The clear dose–response relationships observed for both BMI and BRI strengthen the validity of these indices as indicators of metabolic disease risk, showing that increasing adiposity is associated with progressively higher risks of adverse outcomes. The markedly higher ORs for BRI compared with BMI, particularly for diabetes and metabolic syndrome where differences approached a 2-fold magnitude, emphasize the stronger discriminatory ability of BRI in capturing central adiposity–related risks. The larger differences between BRI and BMI seen in female compared with male may be partly attributed to sex-specific differences in fat distribution and hormonal changes after menopause, which intensify the metabolic consequences of visceral obesity. By contrast, the decline in ORs with advancing age suggests that the predictive power of anthropometric indices diminishes in older populations, likely due to age-related changes in body composition and potential survivorship bias.

The superior performance of BRI observed in our study may also be explained by its closer reflection of visceral adiposity compared with BMI. Whereas BMI is limited by its inability to differentiate fat from lean mass, BRI incorporates waist circumference and height into a continuous measure that reflects both overall adiposity and body shape. Visceral obesity has been shown to exert stronger effects on insulin resistance, chronic inflammation, and atherogenic dyslipidemia than general obesity, which may account for the particularly strong discriminatory ability of BRI for diabetes and metabolic syndrome.

It is important to interpret the markedly higher ORs observed for metabolic syndrome with caution. This may be largely attributable to the fact that abdominal obesity, represented by waist circumference, is one of the diagnostic components of metabolic syndrome. Because BRI inherently incorporates waist circumference into its calculation, a conceptual and statistical overlap exists between the exposure (BRI) and the outcome (metabolic syndrome). As a result, the association between BRI and metabolic syndrome may be inflated, and the magnitude of the observed ORs may not reflect causal strength but rather the shared definitional component.

Previous studies have also examined the relationship between BRI and mortality, reporting a U-shaped association similar to that observed for BMI, where both low and high BRI levels were linked to increased all-cause mortality [14,25]. Although the present study focused on metabolic diseases rather than mortality outcomes, the stronger associations observed between BRI and metabolic diseases—particularly diabetes and metabolic syndrome—are broadly consistent with mortality evidence, as these conditions are major contributors to long-term health outcomes. Nonetheless, caution is warranted in interpreting this correspondence because our cross-sectional design does not allow causal inference regarding mortality risk.

Sex-specific and age-specific differences observed in our analyses may also have biological explanations. The greater predictive value of BRI in female, especially those aged 45 years or older, could be related to hormonal changes around menopause that promote central fat accumulation and exacerbate insulin resistance. Elffers et al. [26] suggested that postmenopausal female may experience more adverse cardiometabolic consequences of visceral obesity compared with male of similar age. Consistent with this, Mousavi et al. [27], in a study of diabetes risk across the female life cycle, compared premenopausal and postmenopausal female and reported that during reproductive age, both BMI and BRI were significantly higher in female with diabetes than in those without, whereas after menopause, only BRI remained significantly different.

The greater discriminatory ability of BRI compared with BMI observed among middle-aged and older male may be attributed to age-related and sex-related differences in body fat distribution. As male age, visceral adiposity tends to increase disproportionately relative to total body weight, while lean muscle mass progressively declines. Because BMI does not distinguish between fat and lean mass, it may underestimate metabolic risk in this population. In contrast, BRI, which incorporates both waist circumference and height into a geometric model, more accurately reflects central obesity and age-related changes in body shape. Therefore, the superior performance of BRI in older male likely reflects its greater sensitivity to visceral fat accumulation, a key determinant of metabolic disease risk.

The limitations of this study are as follows. First, because this study employed a cross-sectional design, causal relationships between variables cannot be established. Thus, the findings should be interpreted with caution, and further cohort studies are necessary to clarify the temporal and causal pathways underlying the observed associations. Second, several confounding variables could not be controlled for: variables related to family history were excluded due to substantial missing data, and those related to dietary habits or nutritional status were removed because of the possibility of reverse causation. Nevertheless, the comparison between BMI and BRI was considered fair, as both indices were analyzed under the same conditions.

Despite these limitations, this study has several strengths. To our knowledge, it is the first investigation in a Korean population to comprehensively assess the discriminatory ability of BRI across multiple metabolic diseases. The use of nationally representative data minimized sampling error and strengthened statistical precision. Additionally, stratified analyses by sex and age enabled the identification of subgroups in which BRI may serve as a particularly effective screening indicator, providing important insights for targeted public health interventions.

Our results suggest that BRI may offer complementary value to BMI as an anthropometric index for identifying individuals at higher risk of metabolic diseases in Korea. Given its ease of calculation, BRI could feasibly be incorporated into routine health examinations, workplace health programs, and community-based screening initiatives to supplement existing indicators. Its relatively stronger performance in female and in middle-aged and older adults also highlights the potential importance of sex-specific and age-specific strategies in metabolic disease prevention. Integrating BRI as a complementary metric within nationwide health surveillance systems, such as KNHANES or the NHIS health checkup program, may help strengthen early detection and prevention efforts.

Furthermore, to enhance the clinical and public health utility of BRI, it is important to establish disease-specific cutoff values that support effective risk stratification and screening decisions. Although the present study focused primarily on comparing the discriminatory ability of BMI and BRI, future research should work toward identifying optimal BRI thresholds for various metabolic diseases across different demographic subgroups. Such efforts will facilitate the broader use of BRI as a standardized diagnostic and preventive tool.

In conclusion, this study demonstrated that BRI was generally superior to BMI in discriminating and assessing associations with metabolic diseases. The advantage of BRI was particularly evident for diabetes and metabolic syndrome. These findings suggest that BRI could serve as a complementary indicator to BMI, offering additional value in clinical practice and national health monitoring systems.

## Figures and Tables

**Figure f4-epih-47-e2025069:**
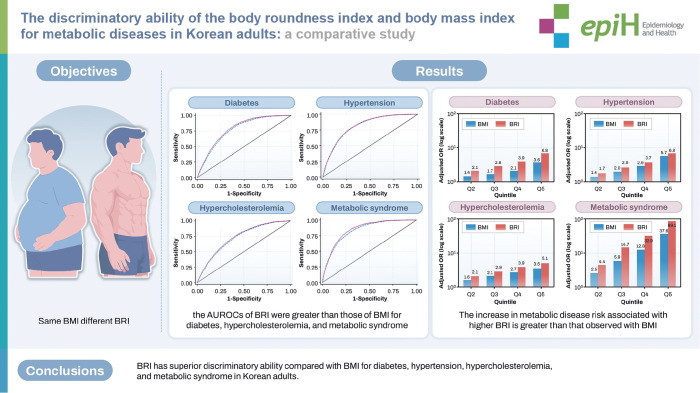


**Table 1.** Demographic characteristics and BMI and BRI status of the study population

Characteristics	Participants	BMI	p-value	BRI	p-value
Total	86,458 (100)	23.9±0.02		3.42±0.01	
Age (yr)	45.9±0.11		<0.01		<0.01
<45	34,745 (49.2)	23.6±0.03		3.01±0.01	
45 to <65	30,799 (35.8)	24.2±0.02		3.63±0.01	
≥65	20,914 (15.0)	23.9±0.03		4.24±0.01	
Sex			<0.01		<0.01
Male	40,527 (52.5)	24.4±0.02		3.45±0.01	
Female	45,931 (47.5)	23.2±0.02		3.38±0.02	
Education status			<0.01		<0.01
≤High school	53,236 (60.9)	24.0±0.02		3.57±0.01	
≥University	28,457 (39.1)	23.7±0.27		3.16±0.01	
Household income (million KRW/mo)			0.22		<0.01
<2	26,012 (24.6)	23.9±0.03		3.74±0.14	
2 to <5	37,799 (46.7)	23.9±0.24		3.34±0.01	
≥5	21,716 (28.7)	23.8±0.33		3.27±0.01	
Physical activity (METs-min/wk)			0.46		<0.01
<600	46,710 (53.0)	23.9±0.02		3.54±0.01	
≥600	39,748 (47.0)	23.8±0.02		3.28±0.01	
Smoking status			<0.01		<0.01
Never	47,859 (53.6)	23.5±0.02		3.37±0.01	
Past	15,671 (19.3)	24.4±0.03		3.58±0.01	
Current	20,506 (27.1)	24.2±0.03		3.38±0.01	
Alcohol consumption			<0.01		<0.01
<1 drink/mo	38,000 (40.6)	23.8±0.03		3.55±0.01	
1-4 drinks/mo	27,176 (35.4)	23.7±0.03		3.23±0.01	
>2 drinks/wk	18,863 (24.0)	24.2±0.03		3.46±0.01	
Metabolic disease					
Diabetes mellitus			<0.01		<0.01
No	73,164 (90.0)	23.7±0.02		3.32±0.01	
Yes	9,944 (10.0)	25.4±0.05		4.34±0.02	
Hypertension			<0.01		<0.01
No	59,836 (74.1)	23.4±0.02		3.16±0.01	
Yes	26,279 (25.9)	25.3±0.03		4.16±0.02	
Hypercholesterolemia			<0.01		<0.01
No	67,655 (83.2)	23.6±0.02		3.28±0.01	
Yes	15,375 (16.8)	25.1±0.03		4.07± 0.01	
Metabolic syndrome			<0.01		<0.01
No	61,208 (73.0)	22.9±0.02		3.05±0.01	
Yes	25,250 (27.0)	26.4±0.03		4.40±0.01	

Values are presented as unweighted number (weighted %) or weighted mean±standard error. BMI, body mass index; BRI, body roundness index; METs, metabolic equivalent of task; KRW, Korean won.

**Table 2.** The AUROCs of BMI and BRI for four metabolic diseases

Participants	n	Diabetes		Hypertension		Hypercholesterolemia		Metabolic syndrome	
		BMI	BRI	BMI	BRI	BMI	BRI	BMI	BRI
Total	86,458	0.783 (0.779, 0.788)	0.793 (0.789, 0.797)	0.822 (0.819, 0.825)	0.821 (0.818, 0.824)	0.739 (0.735, 0.744)	0.745 (0.741, 0.749)	0.844 (0.841, 0.846)	0.851 (0.849, 0.854)
Sex-age									
Male	40,527	0.752 (0.746, 0.758)	0.761 (0.755, 0.767)	0.771 (0.766, 0.776)	0.769 (0.765, 0.774)	0.681 (0.674, 0.687)	0.690 (0.684, 0.697)	0.812 (0.808, 0.823)	0.827 (0.823, 0.831)
<45	15,509	0.781 (0.762, 0.800)	0.788 (0.769, 0.807)	0.754 (0.744, 0.764)	0.746 (0.735, 0.757)	0.718 (0.706, 0.731)	0.732 (0.719, 0.744)	0.842 (0.836, 0.849)	0.850 (0.843, 0.856)
45 to <65	14,740	0.644 (0.633, 0.656)	0.659 (0.648, 0.670)	0.680 (0.671, 0.689)	0.675 (0.666, 0.684)	0.620 (0.609, 0.631)	0.632 (0.621, 0.643)	0.773 (0.765, 0.780)	0.794 (0.786, 0.801)
≥65	10,278	0.590 (0.577, 0.604)	0.610 (0.597, 0.624)	0.645 (0.633, 0.656)	0.643 (0.632, 0.654)	0.630 (0.616, 0.644)	0.640 (0.626, 0.654)	0.770 (0.760, 0.779)	0.789 (0.780, 0.798)
Female	45,931	0.806 (0.801, 0.812)	0.817 (0.811, 0.823)	0.867 (0.863, 0.870)	0.866 (0.863, 0.870)	0.788 (0.783, 0.793)	0.790 (0.785, 0.794)	0.860 (0.857, 0.864)	0.867 (0.863, 0.871)
<45	19,236	0.826 (0.806, 0.845)	0.839 (0.820, 0.858)	0.804 (0.789, 0.818)	0.800 (0.785, 0.815)	0.711 (0.694, 0.727)	0.713 (0.696, 0.730)	0.886 (0.878, 0.895)	0.885 (0.877, 0.894)
45 to <65	16,059	0.713 (0.701, 0.726)	0.732 (0.719, 0.744)	0.714 (0.705, 0.723)	0.714 (0.705, 0.723)	0.681 (0.672, 0.690)	0.687 (0.677, 0.696)	0.793 (0.784, 0.801)	0.802 (0.793, 0.810)
≥65	10,636	0.609 (0.595, 0.622)	0.630 (0.616, 0.643)	0.660 (0.648, 0.671)	0.653 (0.642, 0.665)	0.602 (0.590, 0.613)	0.601 (0.589, 0.612)	0.721 (0.710, 0.731)	0.733 (0.723, 0.743)

Values are AUROC (95% confidence interval). AUROC, area under the receiver operating characteristic curve; BMI, body mass index; BRI, body roundness index.

**Table 3.** Association of metabolic diseases in the highest quintile versus the lowest quintile of BMI and BRI

Participants	n	Diabetes		Hypertension		Hypercholesterolemia		Metabolic syndrome	
		BMI	BRI	BMI	BRI	BMI	BRI	BMI	BRI
Total^1^	86,458	3.6 (3.2, 4.0)	6.8 (5.9, 8.0)	5.7 (5.3, 6.2)	6.8 (6.2, 7.4)	3.5 (3.2, 3.8)	5.1 (4.6, 5.6)	37.8 (35.3, 43.2)	89.1 (77.2, 102.8)
Sex-age^2^									
Male	40,527	3.0 (2.6, 3.4)	4.4 (3.8, 5.2)	5.2 (4.7, 5.7)	5.3 (4.8, 5.9)	3.3 (2.9, 3.7)	4.6 (4.0, 5.2)	38.3 (33.8, 43.3)	54.3 (46.7, 63.1)
<45	15,509	10.6 (5.4, 20.7)	18.4 (6.2, 54.8)	9.0 (6.6, 12.4)	7.9 (5.7, 11.2)	3.6 (2.6, 5.1)	5.5 (3.6, 8.3)	105.6 (63.0, 176.9)	283.4 (115.5, 695.2)
45 to <65	14,740	2.4 (2.0, 3.1)	3.5 (2.7, 4.4)	4.4 (3.7, 5.3)	4.9 (4.1, 5.9)	2.4 (2.0, 3.0)	3.1 (2.6, 3.9)	27.4 (21.8, 34.5)	39.6 (31.2, 50.3)
≥65	10,278	2.2 (1.7, 2.8)	3.0 (2.3, 3.8)	4.1 (3.3, 5.1)	4.5 (3.6, 5.5)	2.4 (1.8, 3.2)	2.9 (2.1, 3.7)	24.5 (18.6, 32.1)	35.1 (26.4, 46.1)
Female	45,931	3.5 (2.8, 4.3)	12.2 (8.2, 18.3)	4.4 (3.8, 5.1)	5.8 (4.9, 6.9)	2.6 (2.2, 2.9)	3.8 (3.2, 4.4)	23.1 (19.4, 27.4)	89.8 (65.3, 123.5)
<45	19,236	26.7 (10.2, 69.9)	45.5 (12.8, 161.3)	8.0 (4.8, 13.3)	7.5 (4.2, 13.3)	3.5 (2.4, 5.0)	3.9 (2.7, 5.6)	99.4 (42.0, 235.2)	275.8 (84.4, 900.9)
45 to <65	16,059	3.5 (2.6, 4.6)	6.3 (4.4, 8.9)	4.5 (3.8, 5.4)	5.3 (4.3, 6.3)	2.3 (2.0, 2.7)	3.1 (2.6, 3.7)	20.3 (16.1, 25.6)	37.9 (28.8, 50.0)
≥65	10,636	1.8 (1.4, 2.2)	2.5 (2.0, 3.1)	3.6 (3.0, 4.4)	3.4 (2.8, 4.1)	1.7 (1.4, 2.1)	1.8 (1.5, 2.2)	10.9 (8.9, 13.3)	14.0 (11.3, 17.4)

Values are adjusted odds ratio (95% confidence interval). BMI, body mass index; BRI, body roundness index.

1 Adjusted for sex, age, education, income, physical activity, alcohol, and smoking.

2 Adjusted for age, education, income, physical activity, alcohol, and smoking.
